# Fatty acids, epigenetic mechanisms and chronic diseases: a systematic review

**DOI:** 10.1186/s12944-019-1120-6

**Published:** 2019-10-15

**Authors:** K. González-Becerra, O. Ramos-Lopez, E. Barrón-Cabrera, J. I. Riezu-Boj, F. I. Milagro, E. Martínez-López, J. A. Martínez

**Affiliations:** 1 Institute of Traslational Nutrigenetics and Nutrigenomics, Health Sciences University Center, University of Guadalajara, Guadalajara, Jalisco Mexico; 2 Department of Nutrition, Food Science, Physiology and Toxicology, Centre for Nutrition Research, University of Navarra, Pamplona, Spain; 3 Faculty of Medicine and Psychology, Autonomous University of Baja California, Tijuana, B.C. Mexico; 4 Navarra Institute for Health Research (IdiSNA), Pamplona, Spain; 5 Centro de Investigación Biomédica en Red Fisiopatología de la Obesidad y Nutrición (CIBERobn), Carlos III Health Institute, Madrid, Spain; 6 Department of Molecular Biology in Medicine, Health Sciences University Center, University of Guadalajara, Sierra Mojada 950, 44340 Guadalajara, Jalisco Mexico; 7 Madrid Institute of Advanced Studies (IMDEA Food), Madrid, Spain

**Keywords:** DNA methylation, Obesity, Epigenetic, N-3 fatty acids, Butyrate, Insulin resistance, Metabolic alterations

## Abstract

**Background:**

Chronic illnesses like obesity, type 2 diabetes (T2D) and cardiovascular diseases, are worldwide major causes of morbidity and mortality. These pathological conditions involve interactions between environmental, genetic, and epigenetic factors. Recent advances in nutriepigenomics are contributing to clarify the role of some nutritional factors, including dietary fatty acids in gene expression regulation. This systematic review assesses currently available information concerning the role of the different fatty acids on epigenetic mechanisms that affect the development of chronic diseases or induce protective effects on metabolic alterations.

**Methods:**

A targeted search was conducted in the PubMed/Medline databases using the keywords “fatty acids and epigenetic”. The data were analyzed according to the PRISMA-P guidelines.

**Results:**

Consumption fatty acids like n-3 PUFA: EPA and DHA, and MUFA: oleic and palmitoleic acid was associated with an improvement of metabolic alterations. On the other hand, fatty acids that have been associated with the presence or development of obesity, T2D, pro-inflammatory profile, atherosclerosis and IR were n-6 PUFA, saturated fatty acids (stearic and palmitic), and *trans* fatty acids (elaidic), have been also linked with epigenetic changes.

**Conclusions:**

Fatty acids can regulate gene expression by modifying epigenetic mechanisms and consequently result in positive or negative impacts on metabolic outcomes.

## Introduction

Nutriepigenomics is an emerging scientific area that studies the relationships between nutrition and the epigenetic. In recent years, several studies have focused on the description of different dietary components that can contribute to modify epigenetic processes and consequently, modulate gene expression and metabolic responses. These epigenetic modifications may be associated with the susceptibility to develop non-communicable chronic diseases (NCCD), such as obesity, lipid disorders, insulin resistance (IR), cardiovascular diseases (CVD), type 2 diabetes (T2D), and some types of cancer [1].

Epigenetics is defined as the study of heritable changes in DNA and histones without concomitant alterations in the nucleotide sequence [2, 3]. These modifications can affect gene expression and the phenotype in response to environmental stimuli [2, 4]. The main epigenetic mechanisms include DNA methylation, histone modifications, and non-coding RNAs such as microRNAs (miRNAs), among others [5].

Epigenetic changes are plastic genomic processes that are influenced by endogenous and exogenous factors, and these modifications could be potentially propagated from one generation to the next [6]. Thus, it might be possible to reprogram epigenetic modifications that are associated with an increased disease risk through nutritional or lifestyle changes. In this context, a number of nutritional factors involved in epigenetic modifications have been reported, including methyl donors, amino acids, vitamins and minerals, polyphenols, and other phytochemicals, and fatty acids (FA) [7].

Regarding FA, some studies have demonstrated the effects of n-3 and n-6 polyunsaturated acids (PUFA) on DNA methylation, including specific responses of eicosapentaenoic acid (EPA), docosahexaenoic acid, (DHA), [8] and arachidonic acid (AA) [9]. However, the mechanisms underlying the effects of different types of FA on epigenetic landmarks, are still not completely known. The most extensively studied FA is butyric acid, a short-chain fatty acid produced in the anaerobic colonic fermentation that can act as an inhibitor of histone deacetylases (HDAC) and has been associated with histone deacetylation [10].

In the last years, the profile of FA intake has dramatically changed from diets with high monounsaturated (MUFA) and polyunsaturated fatty acid (PUFA) content, to a Westernized dietary pattern characterized by a high content in saturated fatty acids (SFA) and trans fatty acids (TFA) and poor in n-3 PUFA [11]. This nutritional transition is associated with the rising prevalence of NCCD, which have been recently associated with aberrant epigenetic changes and are now major cause of death worldwide [12].

It is well known that obesity, CVD, IR, T2D, cancer and other NCCD involving multifactorial and genetic interactions [13]. In this context, the study of pathophysiological, genetic and epigenetic processes could help to design new integral strategies for the prevention and treatment of these conditions [14]. Therefore, the objective of the present review is to describe the role of dietary FA in the modulation of epigenetic landmarks in relation to the development of NCCD, and their ability to reverse the epigenetic landscape.

## Methods

**Fig. 1:**
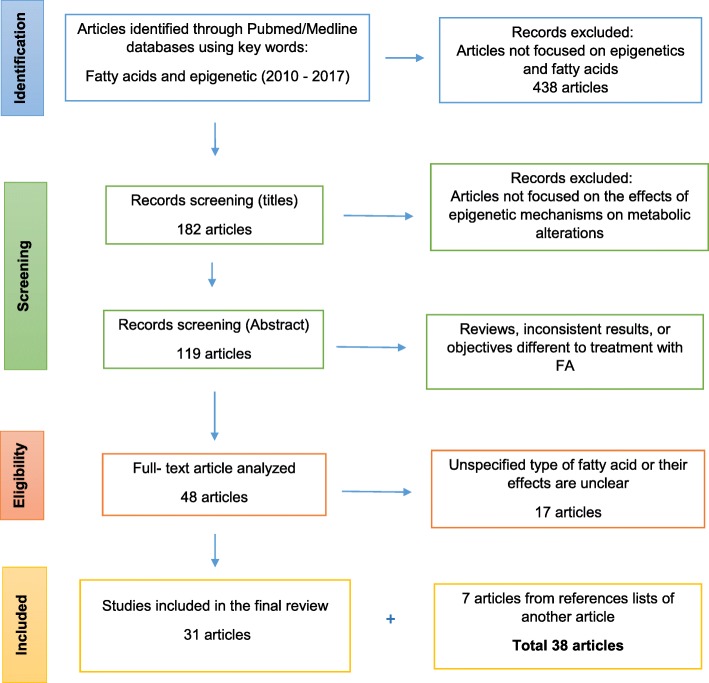
PRISMA flow diagram. Summarizing the selection of papers included in this review (using the term “FA and epigenetics”). Human studies, animal models, and in vitro experiments, were included. FA: Fatty acids

This systematic review has been developed according to the Preferred Reporting Items for Systematic Reviews and Meta-Analyses Protocol (PRISMA-P) guidelines [15]. Literature search was performed using PubMed/Medline databases and just English papers were considered. According to PRISMA-P procedures, the key words “FA and epigenetics” (including SFA, MUFA and PUFA) and the period of publication “2010–2017” were used as filters. At this stage, a total of 620 articles were identified. A flow diagram showing the selection process is depicted (Fig. 1).

### Eligibility criteria

First inclusion criteria were articles analyzing the effects of FA on epigenetics mechanisms. In this section, 438 articles were removed because do not include interactions between epigenetics and FA. In a second step, articles not focused on the effect of metabolic alterations through epigenetic mechanisms in response to FA intake or supplementation were also excluded (*n* = 63). Subsequently, articles showing inconsistent results, did not fulfill quality criteria or using valproic acid (considered a drug), were eliminated (*n* = 71). Lastly, articles that did not specify the type of FA intervention were also excluded (*n* = 17). The final analysis included 31 articles; however, seven additional articles obtained from reference lists were also incorporated in this review (Fig. 1).

### Data extraction, data elements

Relevant information from all 38 papers was analyzed using a standardized data extraction template where two co-authors were specifically involved. Data about the type of FA used for treatment, the study model, the underlying epigenetic mechanism as well as the main results related to metabolic outcomes, were reported, more details are shown in Additional file 1.

### Quality assessment

Study quality was evaluated according to Jadad scale for clinical trials and it was considering randomization, blinding and withdrawals and dropouts. The selection criteria of the articles had to meet at least 3 criteria to be included. In this review, two authors evaluated the quality of papers and to resolve some disagreements all the author make a consensus procedure to define if the article full fill the criteria for be included in the systematic review.

### Data analysis

The acquired information was organized based on the type of FA and experimental model analyzed. The data were sorted according to fatty acids subtypes; firstly, unsaturated fatty acids were described (PUFA: n-3, n-6 and MUFA: oleic) (Table 1). Then, saturated and trans fatty acids were sorted in a second category (Table 2). Butyrate was independently analyzed from other FA because it is a product of anaerobic colon fermentation (Table 3). Finally, articles including more than one type of FA were grouped together (Table 4).

**Table 1:** Effects of unsaturated fatty acids on metabolic outcomes through epigenetic mechanisms

FA	Dose	Study model	Epigenetic mechanisms	Epigenetic signature		Metabolic outcomes	Reference
HUMAN							
PUFA n-3 supplementation	3 g n-3 6-weeks	36 overweight and obese subjects	DNA methylation	286 CpG (93%) 22 CpG (7%)	+ -	Improvement of inflammatory and immune responses, lipid metabolism, cardiovascular signaling, and diabetes pathways, reduction of plasma triglyceride and glucose levels, improved total cholesterol/HDL-cholesterol ratio.	[16]
n-3 intake	93 subjects were in the lowest 3 deciles of PUFA intake and 92 were in the top 3 deciles	185 Yupik/ Alaskan native subjects	DNA methylation	21 CpG 6 CpG	+ -	Improvement of lipid metabolism, insulin sensitivity, glucose tolerance and oxidative stress.	[17]
n-3 supplementation	MedDiet+ OOEV or MedDiet+ nuts	12 subjects of each study group	DNA methylation	With MedDiet + nuts CPT1B/CHKB-CPT1B With MedDiet + OOEV *GNASAS GNAS*	+ -	Benefits in health associated with changes in genes related to intermediate metabolism, diabetes, and anti-inflammatory state.	[18]
n-3 supplementation	6 capsules/ per day n-3 8-weeks	7 overweight and obese women 5 control group	DNA methylation	*CD14, PDK4* and *FADS1* *PDK4* (− 229–227) *CD36* FFAR3 CpG (−18, + 33, and + 77) FFAR3 CpG (− 53 and − 202)	- + + +	Lipid metabolism, improvement of glucose tolerance and diabetes.	[19]
n-6 intake		40 normal-weight women	DNA methylation	*TNF* CpG13 and CpG19 (+ 207 + 317pb)	+	Associated with truncal fat, lipid alterations, TNF-α pathway and inflammation process.	[20]
Transgenerational							
DHA supplementation	400 mg of DHA/day gestation week 18–22 to parturition.	131 pregnant women	DNA methylation	*IGF2* P3 *IGF2* DMR *H19* DMR	- + +	Favors expression of genes involved in growth and development. Decreases the risk to develop obesity (BMI) in infants.	[21]
DHA supplementation	800 mg DHA/day 20 weeks gestation to parturition.	517 pregnant women	DNA methylation	21 DMR		Favors appetite regulation and immune response in infants.	[22]
ANIMAL MODELS							
n-3 supplementation	n-3 1 g/Kg body weight every day for 12 weeks	30 Rats	DNA methylation	% 5mC	+	Anti-colorectal cancer effect.	[23]
n-3 supplementation	34.9% weight as fat, 60% kcal was fish oil for 14 weeks	12 Rats	DNA methylation, Histone methylation and acetylation	NE on methylation Histone H3	++	Ameliorates leptin resistance, decreases accumulation of adipose tissue, regulating food intake and energy expenditure.	[24]
n-3 supplementation	EPA and DHA 0.5% Gromega, pregnant pigs (150 days) and their offspring (lactation 21 days and nursery 56 days)	5 Pigs	DNA methylation and miRNAs	Chromosome 4 DMR Intragenic region chromosome 4 and 12	- +	Improvement of immune response, inflammation, glucose uptake, apoptosis, endoplasmic reticulum stress, insulin resistance, lipid metabolism and oxidative stress.	[25]
IN VITRO MODELS							
n-6 AA	1 μM 10 μM and 100 μM	Human THP-1 monocytes	DNA methylation	Dose-dependent DNA methylation A 10.5% increase in 5mC content at 100 mM compared to 1 μM dose	+	Associated with atherosclerosis, diabetes, inflammatory profile, obesity and cancer	[26]
AA	3 μM	Human umbilical vein endothelial cells (HUVECs) and endothelial progenitors (EPCs)	DNA methylation	Promoter region of genes KDR and Notch4	–	Associated with changes in expression of genes implicated in carcinogenesis and angiogenesis.	[9]
MUFA							
Oleic acid	1 μM 10 μM and 100 μM	In vitro human THP-1 monocytes	DNA methylation	Global hypomethylation at 100 μM compared to the 1 μM dose	–	Anti-inflammatory effects.	[26]
Oleic acid	1–200 μM range	20 pregnancy mice and THP-1 cells	DNA methylation	1–50 μM but in 5 μM weaker response peaking	+	Improvement of proinflammatory profile and adipogenesis	[27]

*FA* Fatty acids, *PUFA* Polyunsaturated fatty acids, *n-3* linolenic acid, *DHA* Docosahexaenoic acid, *EPA* Eicosapentaenoic acid, *AA* Arachidonic acid, *MUFA* Monounsaturated fatty acid, *TNF* Tumor necrosis factor

*DMR* Differentially methylated regions

*NE* No-effect on DNA methylation

**+** hypermethylated

**-** hypomethylated

**++** Hyperacetylation

**Table 2:** Effects of saturated and trans FA on metabolic outcomes through epigenetic mechanisms

FA	Dose	Study model	Epigenetic mechanism	Epigenetic signature		Metabolic outcomes	Reference
HUMANS							
Trans FA							
Industrial TFA	10.2 g/2500 kcal, 3.7% of daily energy	9 healthy men	miRNAs	5 miRNAs in purified HDLs 13 HDL-carried miRNAs to the plasmatic miRNA pool	↑ ↑	Related to carcinogenesis, FA biosynthesis and alteration in FA metabolism	[28]
ANIMAL MODELS							
Transgenerational							
Elaidic acid		20 pregnancy mice and THP-1 cells	DNA methylation	1–50 μM 5.2% increase in 5mC up to 200 μM	+ -	Favors the accumulation of adipose tissue, obesity, and hepatic steatosis	[27]
IN VITRO MODELS							
SFA							
Palmitic acid	750 μM palmitate	In vitro urinary human podocyte cell line and male Sprague-Dawley rats	Histone methylation and acetylation	H3K27me3 and H3K36me2 on promoter region of FOXO1	↓	Related to insulin resistance and decrease of glucose tolerance, favors gluconeogenesis.	[29]
Palmitic acid	1 mM palmitate	In vitro human pancreatic islets	DNA methylation	4561 sites increased DNA methylation (2753 unique genes and 1429 intergenic sites) 129 sites decreased DNA methylation (99 unique genes, and 30 intergenic sites.	+ -	Associated with insulin resistance, lipotoxicity, T2D, glycolysis, gluconeogenesis, dysregulation in FA metabolism related to obesity.	[30]
Palmitate	0.4 mmol/l palmitate	Pancreatic beta cell line and diabetic rats	DNA methylation	No changes in DNA methylation		No change in DNA methylation of *Ins1* promoter under normal or high glucose conditions	[31]
Oleato-palmitate	250 μM oleate-palmitate ratio 1:1	Human skeletal muscle cells from severely obese women	DNA methylation	PPARδ (sites - 71 and 61 bp)	+	Changes in methylation of *PPARD*, increases FA uptake and oxidation, favors abnormal accumulation of lipids in oxidative tissues.	[32]
Stearate and palmitate	3.75 mM. Stearate-palmitate ratio 4:1	Raw264.7 macrophage cell line	DNA methylation	PPARg promoter	+	Promote metabolic disorders and inflammation, increase insulin resistance and obesity.	[33]

*FA* Fatty acids, *TFA* Trans fatty acids, *FA* Fatty acids, *THP-1* Human monocytic cell line, *HDL* High density lipoprotein

↑ Increase

↓ Decrease

**+** hypermethylated

**-** hypomethylated

**Table 3:** Effects of SCFA on metabolic outcomes through epigenetic mechanisms

SCFA	Dose	Study model	Epigenetic mechanism	Epigenetic signature		Metabolic outcomes	Reference
ANIMAL MODELS							
Sodium butyrate	500 mg/kg/day	Juvenile diabetic rats	Histone acetylation	Decreased HDAC activity	↓	Decreases plasma glucose, HbA1c, and beta-cell apoptosis. Favors insulin sensitivity and glucose homeostasis.	[34]
Sodium butyrate	5% NaB (wt/wt)	C57BL/6 J mice	Histone modifications	Modify chromatin structure and repositioning of the −1 nucleosome		Modifies gene expression to have anti-obesity and anti-diabetic effects, improves insulin sensitivity	[35]
Butyrate	1.5 g/kg feed for 21 days	308 chickens	Histone acetylation	Hepatic histone H2A at lysine 5	++	Improves body weight, regulation of cell function	[36]
Sodium butyrate	1% butyrate sodium	Offspring of Sprague Dawley rats	Histone acetylation	Increase of acH3K27 in *Pparg* gene Increase of acH3K9 and acH3K27 on the promoters of C/EBPβ and FAS genes	↑ ↑	Maternal butyrate supplementation during gestation and lactation leads to insulin resistance and accumulation of ectopic lipids, risk Aof development T2D.	[37]
IN VITRO MODELS							
Butyric acid	3 mM of butyrate	Chinese hamster ovary cells	DNA methylation	Around 8113 and 8616 DMR Around 5589 and 6524 DMR	- +	Cell cycle, apoptosis, signaling, protein transport and RNA processing.	[38]
Butyrate	10 mM of butyrate	Bovine cells	Histone modification	Histone H3 and H4	++	Activation of genes related to growth, proliferation, energy metabolism, cell growth and division, cell cycle, apoptosis and differentiation.	[39]
Sodium butyrate	10 mM of NaB	HeLa 57A and HEK293 cells	Histone acetylation	Histone H3K9, H4K5, and H4K16	++	NF-κB activation in response to TNF-α, increased pro inflammatory response and immune responses, cell proliferation and differentiation.	[40]
Sodium butyrate	0.5 mM, 1 mM, 2.5 mM and 5 Mm of NaB	Two human prostate cancer cell lines (LNCaP, C4–2) and one normal prostate cell line (RWPE-1)	Histone acetylation	Lysine 8 and Lysine 12 of Histone H4	++	Suppression of tumor growth in prostate cancer.	[41]
Sodium butyrate	2 μM of NaB	9 human gastric cancer cell lines (AGS, KatoIII, MKN28, MKN45, MKN74, NCI-N87, SNU-1, SNU-16, and NCI-N87	Histone acetylation	Demethylation and histone modification at the promoter of *SFRP1/2*	–	Demises proliferation of human gastric cancer cells (protective effect against cancer),	[42]
Sodium butyrate	5 mM of butyrate	Rat vascular smooth muscle cells (VSMCs) isolated from thoracic aortas	modification of histone H3 by acetylation, phosphorylation and methylation	H3Lys9 H3Lys9, H3Lys4 di-methylation	++	Atheroprotective and antiatherogenic effect, altering G1-specific cell cycle proteins through its chromatin remodeling activity to arrest VSMCs proliferation.	[43]
Combination of butyrate + DHA	5 mM NaB + 50 μM of DHA	In vitro human colon cancer cells	DNA methylation histone acetylation	Reduced methylation of proapoptotic (*BCL2L11*, *CIDEB*, *DAPK1*, *LTBR*, and *TNFRSF25)* genes	**–**	Induction of proapoptotic genes related to cancer.	[44]

*SCFA* Shot chain fatty acids, *FA* Fatty acids, *HDAC* Histone deacetylases, *HbA1c* Glycated hemoglobin, *T2D* Type 2 Diabetes, *NFκB* Nuclear factor kappa B

++ hyperacetylation

↑ Increase

↓ Decrease

+ hypermethylated

**-** hypomethylated

**Table 4:** Comparison of different types of FA influences on epigenetic mechanisms

FA	Dose	Study model	Epigenetic mechanism	Epigenetic signature	Metabolic effect	Reference
HUMANS						
Excessive SFA palmitic acid intake (+ 750 kcal/d) Excessive PUFA n-6 intake (+ 750 kcal/d)	High-caloric muffins that contained refined palm oil or refined sunflower oil for 7 wk	17 subjects (adipose tissue) 14 subjects (adipose tissue)	DNA methylation	PUFA n-6 + SFA modify 4933 CpG sites (4795 hypermethylated and 138 hypomethylated) Expression changes in 1117 transcripts (776 up, 241 down regulated) 26 pathways up-regulated 3 pathways down-regulated	SFA and PUFA n-6 diets modify methylation patterns of genes related to adipose tissue accumulation, obesity, pathways related to cancer, cell cycle, FA uptake, transport, and lipid metabolism.	[45]
Lower PUFA/SFA ratio and lower PUFA+MUFA/ SFA ratio	A higher unsaturated: saturated ratio considered ‘healthier’, and a lower unsaturated: saturated ratio considered ‘unhealthier‘	35 obese and 34 normal-weight preadolescents	DNA methylation	The methylation levels of 2 islands, 11 island shores, and 16 sites were correlated with PUFA/SFA; 9 islands, 26 island shores, and 158 sites for MUFA/SFA; 10 islands, 40 island shores, and 130 sites for (MUFA+PUFA)/SFA	A lower PUFA/SFA ratio was associated with adipogenesis and mechanisms regulated by PPARα, regulation of energy intake, inflammatory processes and obesity; a lower MUFA+PUFA vs SFA ratio was related to pathways linked to NF-kB (inflammation process)	[46]
Fish oil (FO) and Sunflower oil (SO)	3.8 g/day of fish oil (FO) or sunflower oil (SO) for 9 months	12 (9-months-old) infants	DNA methylation	Change in the methylation profile (> 10%) of 43 CpG	FO supplementation was associated with higher amounts of n-3, EPA, and DHA and lower levels of n-6 and AA in RBC, improved arterial pressure and a tendency to lower levels of IL-6.	[47]
PUFA (EPA) MUFA (palmitoleic acid) SFA (palmitic acid)	A single Western diet (post-prandial) or no meal (fasting samples).	49 lactating infants and 12 adult males	DNA methylation Histone deacetylation	Global DNA methylation was higher in PUFA than in MUFA and SFAs.	SFA were associated with obesity (BMI), lipid metabolism, and glucose disbalance, whereas PUFA (EPA) were related to normal weight, and MUFA with insulin sensitivity.	[48]
ANIMAL AND IN VITRO MODELS						
PUFA Linoleic acid (olive oil) MUFA Oleic acid (sunflower oil) SFA palmitic acid (coconut oil)	10% fat of different oils	24 rats/3 T3-L1 cells	DNA methylation	Hypomethylation in *Tnf* promoter in SFA vs PUFA and MUFA	SFA was associated with inflammation (TNF-α elevation), adiposity and obesity, whereas PUFA and MUFA did not induce changes in TNF-α	[49]
PUFA Linoleic n-6 (sunflower oil) MUFA oleic FA (olive oil) SFA palmitic FA (coconut oil)	10% fat of the different oils	Rats / 3 T3-L1 cells.	DNA methylation	Lower methylation levels of *Vegfb* promoter in rats that were fed with coconut oil vs olive and sunflower oil	SFA was related to higher levels of Vegfb, involved in insulin resistance, lipid distribution and lipid metabolism in type 2 diabetes vs MUFA and PUFA	[50]
High fat butter (SFA) Fish oil (FO) (n-3 PUFA)	Rats received 3.5, 7% or 21% butter or fish oil (FO) from 14 days preconception until weaning	6 rats per group offspring	DNA methylation	Methylation of CpG (− 623,− 394, −84 and − 76) of *Fads2* was higher in the offspring of dams fed 21% than 3.5% or 7% fat; FO higher than butter.	SFA was associated with fat accumulation in liver, dysregulation of vascular tone vs n-3 PUFA. Epigenetic regulation of *Fads2* may contribute to the regulation of PUFA synthesis.	[51]
Olive oil (OO) Fish oil (FO) Linseed oil (LO) Palm oil (PO)	80–90 mg/day from conception to day 12 of gestation	Pregnant rats and their offspring	miRNAs	Pregnant rats fed SO and FO diets showed a significant lower expression of miR-449c-5p, miR-134–5p, miR-188, miR-32, miR130a, miR-144–3p, miR-431, miR-142–5p, miR-33, miR-340–5p, miR-301a, miR-30a, miR-106b, and miR-136–5p, as compared with OO, LO, and PO diets in liver and adipose tissue.	Adipose tissue mass was lower in the FO group compared with other groups, except with LO group. Decreased expression of miRNAs related to insulin and glucose metabolism compared with PO and OO No differences in miRNA expression between FO and LO	[52]

## Results

### Unsaturated fatty acids

#### Human studies

##### N-3 PUFA

In the last years, many investigations have focused on the effects of n-3 PUFA in the prevention and treatment of different metabolic alterations. Thus, Tremblay and collaborators investigated the effect of n-3 PUFA supplementation in overweight and obese subjects on epigenetic modifications [16]. They found that after a 6-month supplementation 308 CpG sites (231 genes) had different methylation pattern, of which 286 CpG sites were hypermethylated representing 93% of the changes after the supplementation and 22 were hypomethylated (just 7%), using ingenuity pathway analysis system it was reported these epigenetic changes were related to pathways associated with inflammatory and immune responses, lipid metabolism, T2D, and cardiovascular signaling [16].

Another study in obese subjects under an energy-restricted diet supplemented with n-3 PUFA-rich fish oil conducted by do Amaral and collaborators found that the methylation levels of *PDK4 (*Pyruvate Dehydrogenase Kinase 4) CpG sites − 222 and − 50 and *FADS1* CpG − 25 − 22 − 20 were increased in the group supplemented with fish oil. Furthermore, n-3 PUFA supplementation was accompanied by improved weight loss, which was associated with changes in the methylation pattern of one specific CpG site in *CD36*, a gene that encodes a membrane glycoprotein that plays a relevant role in lipid metabolism and may be implicated in obesity-related complications like glucose intolerance and T2D [19].

On the other hand, Aslibekyan et al. investigated the effect of n-3 PUFA intake in a population of Yupik natives, considering that this population had a higher intake of fish-derived n-3 PUFA [17]. For this study, the population was categorized in higher and lower deciles of a nitrogen stable isotope ratio (δ15N), which is a biomarker of n-3 PUFA intake and thus, n-3 PUFA plasma content. The authors found 27 differentially methylated CpG sites at biologically relevant regions that reached epigenome-wide significance and highlighted that DNA methylation may reduce *FAS* (apoptosis antigen 1) expression and, consequently, regulate lipid metabolism through the apoptotic pathway. Also, the methylation pattern of *AHRR (*Aryl-Hydrocarbon Receptor Repressor), a gene that is involved in oxidative stress, was affected by the n-3 PUFA intake, which was accompanied by a positive impact on glucose tolerance and insulin sensitivity [17].

In addition, Arpón et al. studied the effect of Mediterranean Diet (MedDiet) complemented with extra virgin olive oil (EVOO) or nuts on DNA methylation within PREDIMED (PREvención con DIeta MEDiterránea) study. They compared the two diets MedDiet + EVOO and MedDiet + nuts with a low-fat control group during five-year follow-up and found that MedDiet + nuts favors a hypermethylation of cg01081346 in CPT1B/CHKB-CPT1B genes (Carnitine palmitoyltransferase 1B/Choline kinase-like, Carnitine palmitoyltransferase 1B) and MedDiet + EVOO induce hypomethylation in cg17071192 in GNAS/GNASAS genes *GNAS/GNASAS* (Guanine Nucleotide Binding Protein, G Protein), however both diets were associated with intermediate metabolism as well as improve genes involved in diabetes and inflammation pathways [18].

##### Transgenerational studies

Several investigations have reported the effects of n-3 PUFA supplementation (DHA) in the maternal diet on epigenetic changes in the offspring [21, 22]. Thus, Lee and collaborators demonstrated that DHA supplementation in pregnant women demonstrated higher methylation levels of *IGF2/H19* in their offspring versus control group, a gene that is crucial for the correct fetal growth, development, and metabolism of the infants and this effect was dependent on the maternal BMI before pregnancy. Furthermore, *IGF2*/*H19* DMR methylation changes have also been associated with paternal obesity or the risk of overweight, diabetes or some types of cancer in early life [21].

On the other hand, Van Dijk et al. found in a large randomized controlled trial that DHA supplementation during pregnancy did not significantly affect the global methylation pattern, although they identified 21 differentially methylated regions (DMRs) at birth (this difference was sex-dependent) in genes implicated in diverse functions including lipid exchange between membranes (*ESYT3*), appetite regulation (*CCK*), and immune function (*RAET1L* and *LTB*) among others [22].

##### N-6 PUFA

A trial by Hermsdorff et al. showed that AA intake (an n-6 PUFA) was related with higher values of truncal fat, BMI, and waist circumference in women [20]. Moreover, they found a negative correlation between the methylation of *TNF* and the levels of this proinflammatory cytokine. In particular, the hypermethylation of two CpGs of this gene (+ 207 and + 317pb) was associated with the under expression of the gene, and the result of a linear regression model suggest this methylation levels of TNFα promoter were associated with n-6 PUFA intake, suggesting a complex nutriepigenomic interaction that could exacerbate the proinflammatory state [20].

#### Animal models

##### N-3 PUFA

In a study to explore whether n-3 PUFA affects DNA methylation levels in colorectal cancer, rats were fed with n-3 PUFA during tumor induction [23]. The main results showed that the tumor incidence in rats fed the n-3 PUFA-enriched diet was lower than in the non-treated group, demonstrating that the anti-tumorigenic effect of n-3 PUFA was mediated by an increase of DNA methylation [23]. On the other hand, Shen et al. observed that n-3 PUFA could modulate histone modifications by inhibiting enzymes that catalyze or alter the availability of substrates that are required for enzymatic reactions. A significantly lower activity of DNMT1 (DNA methyltransferase 1) and MBD2 (Methyl-CpG-binding domain protein 2) enzymes was observed in mice fed the n-3 PUFA-enriched diet. This outcome was accompanied by an increase in H3 acetylation, lower binding levels of HDAC1, HDAC2, HDAC6 and higher levels of methyl-H3K4 and -H3K9. Hence, the authors concluded that the regulation of leptin expression by n-3 PUFAs is mediated by epigenetic factors, such as MBD2 and histone modifications. Furthermore, n-3 PUFA supplementation in high fat-fed rodents decrease leptin mRNA expression, ameliorate leptin resistance, and decreased the differentiation and proliferation of adipocytes and their storage capacity [24].

##### Transgenerational studies

A genome-wide methylation study was conducted in pigs to determine the effect of prenatal and postnatal n-3 PUFA supplementation (throughout gestation, lactation, and post-weaning periods) on the methylation pattern of the offspring [20]. Different methylation patterns were observed when comparing the supplemented and non-supplemented groups in chromosome 4, finding a hypomethylated DMR in supplemented groups; conversely, hypermethylation was detected in two intergenic regions of chromosomes 4 and 12. The authors concluded that the genes differentially methylated in the offspring were mainly involved in pathways that were improved by the n-3 PUFA supplementation, such as apoptosis, endoplasmic reticulum stress, glucose and insulin homeostasis, immune function, inflammatory profile, glucose uptake, lipid metabolism, and oxidative stress [25]. On the other hand, another study reported that EPA was able to inhibit the expression of lipogenic genes while up-regulating genes involved in fatty acid oxidation [53].

In conclusion, the effects of PUFA depend upon the subtype of FA, (n-6 or n-3; AA and EPA-DHA, respectively), the doses, the sources, and the way of administration (in foods or as nutraceutical). In this sense, more studies are needed in humans and animal models, to uncover the epigenetic effects of PUFA in relation to their beneficial role in NCCD.

#### In vitro models

##### N-6 PUFA

Silva-Martínez et al. studied the effect of AA on global DNA methylation and gene expression in cultured human THP-1 monocytes. The cell stimulation was for 24 h using different concentrations of AA (1, 10, or 100 μM). The results showed that AA induced a dose-dependent DNA hypermethylation peak at the 100 mM dose and the AA stimulation could alter the methylation profile in a similar way that was reported with palmitic acid (saturated acid that was related with aberrant epigenetic changes). This methylation profile was associated with the alteration of pathways involved in metabolic diseases like atherosclerosis, T2D, obesity, the proinflammatory profiles, and some types of cancer [26].

Another research group employed human umbilical vein endothelial cells (HUVECs) and endothelial progenitors (EPCs) to study the effect of AA on DNA methylation and the expression of genes related to angiogenesis as a mechanism involved in the carcinogenesis process [9]. After the stimulation with AA (3 μM) for 24 h, the expression of 18 proangiogenic genes was affected. The authors concluded that the beneficial effect of AA on carcinogenesis may be due, at least in part, to changes in the expression of angiogenic genes, which may be mediated by changes in DNA methylation [9].

##### MUFA

In addition to AA, Silva-Martínez et al. also evaluated the impact of oleic acid (OA) on cultured human THP-1 monocytes [26]. This study demonstrated that OA had an opposite effect than AA, inducing a global hypomethylation and consequently an expression pattern that were related with an improvement of the inflammation profile [26].

As previously described for PUFA, the epigenetic effects of MUFA depended on the subtype of FA and the doses. For example, OA, whose principal sources are vegetable, oils like olive oil, can ameliorate processes related to atherosclerosis, inflammation, T2D and obesity through epigenetic modifications [26].

By other hand, palmitoleic acid is an n-7 MUFA that is biosynthesized from palmitic acid (SFA) whose principal sources are of animal origin and dairy products [54]. Thus, FA can also alter the epigenome, affecting genes associated with prevention of insulin resistance and diabetes and improved lipid and glucose metabolism [55].

### Saturated and trans fatty acids

#### Human studies

##### Trans FA

Dietary trans-fatty acids (TFA) are associated with an increased risk of metabolic diseases. Some of these effects can be mediated by epigenetic mechanisms. For example, a study in humans associated industrial TFA consumption with HDL-carried miRNA concentrations and plasmatic HDL-c levels [28]. The diet rich in industrial TFA altered the concentrations of 5 miRNA in purified HDL and also contributed to 13 HDL-carried miRNA to the plasmatic miRNA pool. These miRNAs modified through the TFA- enriched diet were associated with lipid metabolism and extracellular matrix receptor interaction, suggesting an important role of miRNAs in plasma lipid metabolism regulation [28].

### Animal models

#### Trans FA

##### Transgenerational

Flores-Sierra et al. studied the effects of elaidic acid (EA) supplementation during either pregnancy or lactation in C57BL/6 mice. In both cases, EA supplementation was observed to induce global DNA methylation in the adipose tissue of the offspring 3 months after birth, and it was associated with weight gain and adipose tissue accumulation [27].

#### In vitro models

##### Trans FA

The study by Flores-Sierra et al. evaluated the effect of TFA elaidic acid (EA; tC18:1) on global DNA methylation and gene transcription in cultured human THP-1 monocytes. They found a biphasic dose-dependent response and global hypermethylation was described in the 1–50 μM concentration range, whereas global hypomethylation was observed in concentrations up to 200 μM. The main results showed that EA affected the expression of genes related to pro-inflammatory and adipogenic profiles, but it also affected DNA methylation, suggesting that EA can target gene-body or intergenic regulatory elements [27].

##### Saturated FA

Investigations carried out by Kumar and et al. studied models of IR in human-urine derived podocyte-like epithelial cells (HUPECs) and in male Sprague-Dawley rats, which were fed a high-fat diet. HUPECs were stimulated with 750 μM palmitate, a concentration that is two to three times higher than the palmitate level in normal non-esterified fatty acids (NEFA) [29]. The results showed that an excess of circulating palmitate generated a FA–induced metabolic memory possibly by altering the levels of H3K36me2 and H3K27me3 on the *FOXO1* promoter region, increasing its activity. In conclusion, palmitate favored IR-induced gluconeogenesis and hyperglycemia, and this effect persisted even after normalization of lipid levels both in vitro *and* in vivo, representing cellular metabolic memory [29].

In this context, other researchers have analyzed the effect of palmitate on genome-wide mRNA expression and DNA methylation, in human pancreatic islets in vitro [30]. They reported an increase in the average global DNA methylation in different gene regions including TSS1500, 5′UTR, gene body, 3′UTR and intergenic regions, and a small decrease in global methylation in TSS200 and the 1st exon. Palmitate altered DNA methylation levels in 290 genes, 73 of which were related to BMI. The expression of 1860 genes were also affected by palmitate, including genes involved in T2D (*TCF7L2*, *GLIS3*, *HNF1B,* and *SLC30A8*), and genes associated with glycolysis and gluconeogenesis, FA metabolism dysregulation, and one carbon pool by folate [30].

On the other hand, Ishikawa et al. differed from the other reports, finding that palmitate did not affect DNA methylation levels of the *Ins1* gene promoter in normal or high glucose conditions, which could be due to differences in the doses and genes considered in both studies [31].

Furthermore, Maples et al. found that oleate-palmitate (250 μM oleate-palmitate 1:1 ratio) favored DNA methylation in relation to PPAR δ expression in human skeletal muscle cells (HSkMC) from lean and severely obese women. However, this increase in DNA methylation was lower in HSkMc from obese women, suggesting that obesity can activate transcriptional regulators of FA oxidation in response to FA exposure. In conclusion, the occurrence of different epigenetic alterations in HSkMC after lipid stimulation suggests that a specific epigenetic programming may occur in obese subjects as a response to their own environmental conditions [32].

The effects of stearate and palmitate on the methylation of *Pparg* promoter were investigated in Raw264.7 murine macrophages. The incubation with these SFA increased IL-4 levels and the methylation of *Pparg*, suggesting that *Pparg* hypermethylation could mediate the proinflammatory effects of these SFA and contribute to IR in obesity [33].

The harmful effects of some SFA are well known. For example, palmitic and stearic FA have been involved in pro-inflammatory and metabolic alterations. Different investigations have demonstrated their contribution to the modulation of DNA methylation and histone acetylation in relation to their effects on IR, obesity, hyperglycemia, T2D, lipotoxicity, dysregulation of lipid metabolism, and abnormal lipid accumulation [29–33]. In the last years, the increased consumption of processed and industrialized food, with higher amounts of SFA and TFA, has been associated with inflammation, adipogenesis, abnormal accumulation of adipose tissue, alterations in lipid metabolism, and carcinogenesis processes, which could be mediated by changes in DNA methylation, covalent histone modifications, and some miRNAs.

##### Short-chain fatty acids

Short-chain fatty acids (SCFA) are products of microbial fermentation that can be absorbed in the large intestine [56]. These SCFA can modify epigenetic landmarks (i.e., histone acetylation) and modulate the expression of genes related to pathways associated with cancer, lipid metabolism, glucose homeostasis, and insulin sensitivity, among others. For example, sodium butyrate (NaB) has been demonstrated to inhibit HDAC activity [57–59].

#### Animal models

##### Sodium butyrate (NaB)

Research by Khan et al. studied the effect of NaB supplementation in juvenile diabetic rats, demonstrating a role for NaB as an HDAC inhibitor associated with a decrease in glucose and Hba1c, favoring insulin sensitivity and reducing the risk of developing diabetes [34].

Protective anti-obesity and anti-diabetic effects of NaB have been also reported in a model of C57BL/6 J mice exposed to a high-fat diet [35]. NaB prevented the increase of body weight and adiposity and improved insulin sensitivity, increasing the percentage of type-1 fibers and improving acylcarnitine profiles in muscle [35]. In this context, Mátis et al. also found in chickens that NaB improved body weight and favored cell function regulation, which was mediated by epigenetic changes, such as histone hyperacetylation [36].

##### Transgenerational

In a transgenerational study, Huang et al. demonstrated that an unbalanced maternal diet was determinant in the development of IR and obesity in the offspring. Moreover, they analyzed the effect of maternal butyrate supplementation on insulin sensitivity and lipid metabolism in the skeletal muscle of the offspring. The rats received butyrate diet (1% NaB) during gestation and lactation for 60 days. The offspring of dams that were supplemented with NaB had impaired glucose tolerance and a higher HOMA index (insulin resistance), which was associated with an overexpression of lipogenic genes. This was accompanied by an increase in histone H3 (Lys9) and H3 (Lys27) acetylation in relation to lipogenic genes in the skeletal muscle of the adult offspring. The authors concluded that, in this model, butyrate impaired lipid metabolism and insulin sensitivity in the offspring [37]. This negative effect of butyrate was inconsistent with other investigators, suggesting that dose and duration might be important, and indicating that more studies are necessary to elucidate the role of NaB and other SCFA in the prevention or treatment of chronic diseases.

#### In vitro models

Chinese hamster ovary (CHO) cells were used to analyze the effect of NaB on the transcriptome and epigenome. In this study, NaB induced hypomethylation in genes belonging to pathways associated with the cell cycle, signaling and apoptosis, whereas hypermethylation was observed in genes implicated in protein transport and RNA processing. On the other hand, genes related to protein biosynthesis, the differentiation process and RNA metabolism, were both hyper and hypomethylated. Besides, authors hypothesized that the affected gene regions presented regulatory regions closely linked with the cellular response to butyrate stimulation [38].

Another study performed in bovine cells analyzed the effect of NaB supplementation on histone modifications. The main findings of the investigation were that the inhibition of HDAC caused by NaB promoted hyperacetylation of histones and modified the expression of genes associated with cell growth, proliferation, energy metabolism, cell cycle, apoptosis, and differentiation [39].

Likewise, another study found that both, butyrate and propionate were able to increase histone acetylation in HELA and HEK293 epithelial cells, and enhance NF-κB activation (in response to TNF-α) by means of the induction of toll-like receptors (TLRs) These SCFA had an effect on the proinflammatory response, cell proliferation and differentiation, redirection of innate immune response, and cytokine/chemokine expression [40].

Paskova et al. demonstrated that NaB was able to modify the expression of androgen receptors in prostate cancer cells through an increase of H4 (Lys8) and H4 (Lys12) acetylation, favoring the suppression of tumor growth. However, this effect was minimal in normal cells, suggesting a protective role of NaB in the development of prostate cancer mediated by epigenetic modifications [41].

Consistent with this finding, other authors have reported protective effects of NaB in human gastric cancer cells, inducing demethylation and histone modifications at the promoter region of *SFRP1/2*, and restoring SFRP (Secreted Frizzled-Related Protein) expression in human gastric cancer cells. The authors proposed that NaB induced apoptosis, favored complex formation, promoted caspase activation, and blocked the potential of cancer cells [42].

Finally, an in vitro study combining 5 mM NaB plus 50 μM DHA, evaluated histone modification and DNA methylation in genes involved in apoptosis. It was demonstrated that this combination had a hypomethylation effect on proapoptotic genes (*Bcl2l11*, *Cideb*, *Dapk1*, *Ltbr*, and *Tnfrsf25)* and an increase in global H4 histone acetylation in cells treated with NaB combined with DHA; this induction of apoptosis had an anticancer effect [44].

Other authors studied the effects of NaB on histone modifications and its consequence on G1-specific cell cycle regulators in vascular smooth muscle cells (VSMC), trying to explain the interaction between chromatin remodeling and the antiproliferative action of butyrate. In this model, NaB acted as an HDAC inhibitor and caused a reorganization of chromatin, affecting the expression of negative and positive cell cycle regulators and arresting VSMC proliferation. Hence, NaB was considered a possible therapeutic agent against atherosclerosis [43].

The metabolic effects of butyrate are controversial because some studies have reported positive outcomes, such as a reduction in plasma glucose levels and HBA1c, and an improvement in insulin sensitivity and glucose homeostasis, preventing the increase of body weight and adiposity and inducing proapoptotic genes related to cancer. On the other hand, other studies have described negative effects of butyric acid, including IR, increased risk of T2D, lipid accumulation and a pro-inflammatory profile. Hence, more studies are needed to elucidate the metabolic effects of SCFA and the underlying epigenetic mechanisms, such as HDAC inhibition, in order to clarify their role as therapeutic tools against metabolic alterations and chronic diseases.

### Comparison of different types of fatty acids

#### Human studies

In order to analyze the effects of excessive palmitic acid and n-6 PUFA intake, subjects were instructed to continue with their habitual diet just with the addition of an extra high calorie (750-kcal) muffin rich in either palmitic acid (*n* = 17) or n-6 PUFA-rich sunflower oil (*n* = 14). An adipose tissue biopsy was obtained before and after the intervention period (7 weeks). In particular, SFA overfeeding increased the mean methylation of 125 genes and PUFA overfeeding changed the mean methylation of 1797 genes, only 47 genes overlapped between the two diets, which ones were related to adipose tissue accumulation, obesity, FA uptake, transport, and lipid metabolism insulin resistance and inflammation pathways. These results suggest that DNA methylation may be involved in the individual response to FA overfeeding [45].

Voisini et al. studied the impact of different ratios of PUFA, MUFA and SFA in 91 Greek preadolescents (< 10 years). They analyzed the effects of low PUFA:SFA ratio, low MUFA:SFA and low MUFA+PUFA:SFA ratios on genome-wide DNA methylation. The genes altered in the lower PUFA:SFA ratio were associated with adipogenesis, gene regulation by PPARα, regulation of energy intake, the inflammatory process and obesity. The low MUFA+PUFA:SFA ratio was related to pathways linked to NF-kB (inflammation process). These results suggest that different types of FA have different effects on the epigenome, leading to different physiological responses [46].

On the other hand, Lind et al. designed a study encompassing 133 (9 month-old infants) that were supplemented with a teaspoon of fish oil (1.5 g/day n-3, 400 mg DHA and 1100 mg EPA) or sunflower oil (3.8 g/day) during a 9 month period. They analyzed global DNA methylation and did not find statistical differences between groups; however, they reported that 43 CpG had a 10% difference or more in the absolute methylation level between groups, demonstrating differential effects of both FA. In the PUFA group, they found a higher amount of n-3, EPA and DHA, but lower levels of n-6 and AA in red blood cells (RBC), which was associated with an improvement of arterial pressure and a tendency of lower IL-6 levels [47].

Another study including two different human cohorts, lactating infants, and adult men, attempted to assess if there was an association between DNA methylation and different types of FA, in both fasting and the postprandial state. In the postprandial state, the participants received a representative meal of the western diet (hamburger, fries and coke) and blood was taken after the meal consumption and every 2 h until 6 pm. In the fasting day, volunteers were maintained in the fasted state from 10 am until 6 pm and blood was taken every 2 h. Furthermore, the subjects were separated according to BMI in normal-weight, overweight and obese. Results evidenced a different methylation pattern depending on the BMI and the fasting/postprandial state. The study found that DNA methylation and histone deacetylation mediated by PUFA were related to a cardioprotective and normal-weight status, in contrast to epigenetic landmarks modulated by MUFA (palmitoleic acid) and SFA (palmitic acid) that were associated with pathways implicated in obesity, dysregulation of lipid metabolism, and glucose misbalance [48].

#### Animal and in vitro models

A study in 34 rats and 3 T3-L1 cells compared the administration of different types of FA: sunflower oil rich in linoleic acid as PUFA, olive oil rich in oleic acid as MUFA, and coconut oil rich in palmitic acid as SFA. In rats, DNA methylation of the *Tnf* promoter was analyzed in the visceral adipose tissue. While both linoleic acid (PUFA) and oleic acid (MUFA) did not change *Tnf* methylation levels, palmitic acid increased *Tnf* methylation and was associated with inflammation, adiposity, and obesity. The study also concluded that FA may regulate adipocyte TNF-α levels through changes in the methylation levels of the *Tnf* promoter [49].

Moreover, Monastero et al. analyzed the dietary FA-mediated epigenetic regulation induced by the Vascular Endothelial Growth Factor B (VEGF-B) in adipose tissue of rats and in 3 T3-L1 cell lines [50]. Rats fed with coconut oil presented higher levels of VEGF-B expression and levels of protein, which was associated with the methylation levels of the promoter. Rats fed sunflower oil showed the lowest levels of VEGF-B while higher VEGF-B levels were associated with IR and T2D, as well as an impaired lipid metabolism [50].

##### Transgenerational

A trasgenerational trial was designed by Hoilea et al. to determine the effect of maternal FA consumption on the PUFA status and the epigenetic regulation of fatty acid desaturase 2 (Fads2) involved in PUFA synthesis. The dams received two different FA-rich foods, butter (rich in SFA) or fish oil (rich in n-3 PUFA) and afterwards, the offspring were evaluated. They found a negative correlation between *Fads2* expression and the promoter methylation levels. The methylation level of *Fads2* was higher in the fish-oil group that in the butter group, which was related to a higher accumulation of fat in the liver and a dysregulation of the vascular tone in the butter group. In conclusion, the type of FA affected the regulation of the PUFA synthesis through epigenetic mechanisms [51].

The type of FA can also affect other epigenetic mechanisms, such as the expression of miRNAs, which can modulate the expression of different genes [60]. A study in which pregnant rats were fed soybean oil, olive oil, fish oil, linseed oil, or palm oil diets from conception to day 12 of gestation, the aim was to analyze miRNA expression in adipose tissue and liver of dams and their offspring. The adipose tissue mass was lower in the fish oil and linseed oil groups compared with other groups. Some hepatic miRNAs, such as miR-192–5p, miR-10b-5p, miR- 377–3p, miR-215, miR-21–5p and mir-26b- 5p, were downregulated by fish oil compared with olive oil and palm oil diets. These miRNAs are involved in insulin homeostasis and glucose metabolism. This study concluded that the maternal intake of diverse types of FA during pregnancy can modulate miRNA expression in both maternal and offspring tissues, relating to epigenetic mechanisms and phenotypic outcomes in the adult offspring [52]. Other studies found that a high-fat diet in pregnancy and lactation modulated hepatic miRNAs in the offspring [61, 62]. Hence, it is necessary to design more studies to clarify the role of FA in the modulation of miRNA expression and its association with metabolic alterations.

## Conclusions

Over the last years, a growing number of investigations have been focused on the protective/beneficial effects of different FA, including n-3 PUFA and SCFA, in NCCD. The most consistent literature shows that some of these effects could be mediated by epigenetic mechanisms which play a role in the regulation of gene expression. In addition to n-3 PUFA and SCFA, other FA types, such as n-6 PUFA, MUFA, SFA, and TFA may also alter epigenetic mechanisms, and their effects are still under research. The results show that different types of FA have a different effect on the epigenome, mainly on DNA methylation; however, it is necessary to perform more studies focused on other epigenetic mechanisms, such as histone modifications and miRNAs and their subsequent effects on the regulation of gene expression.

**Fig. 2:**
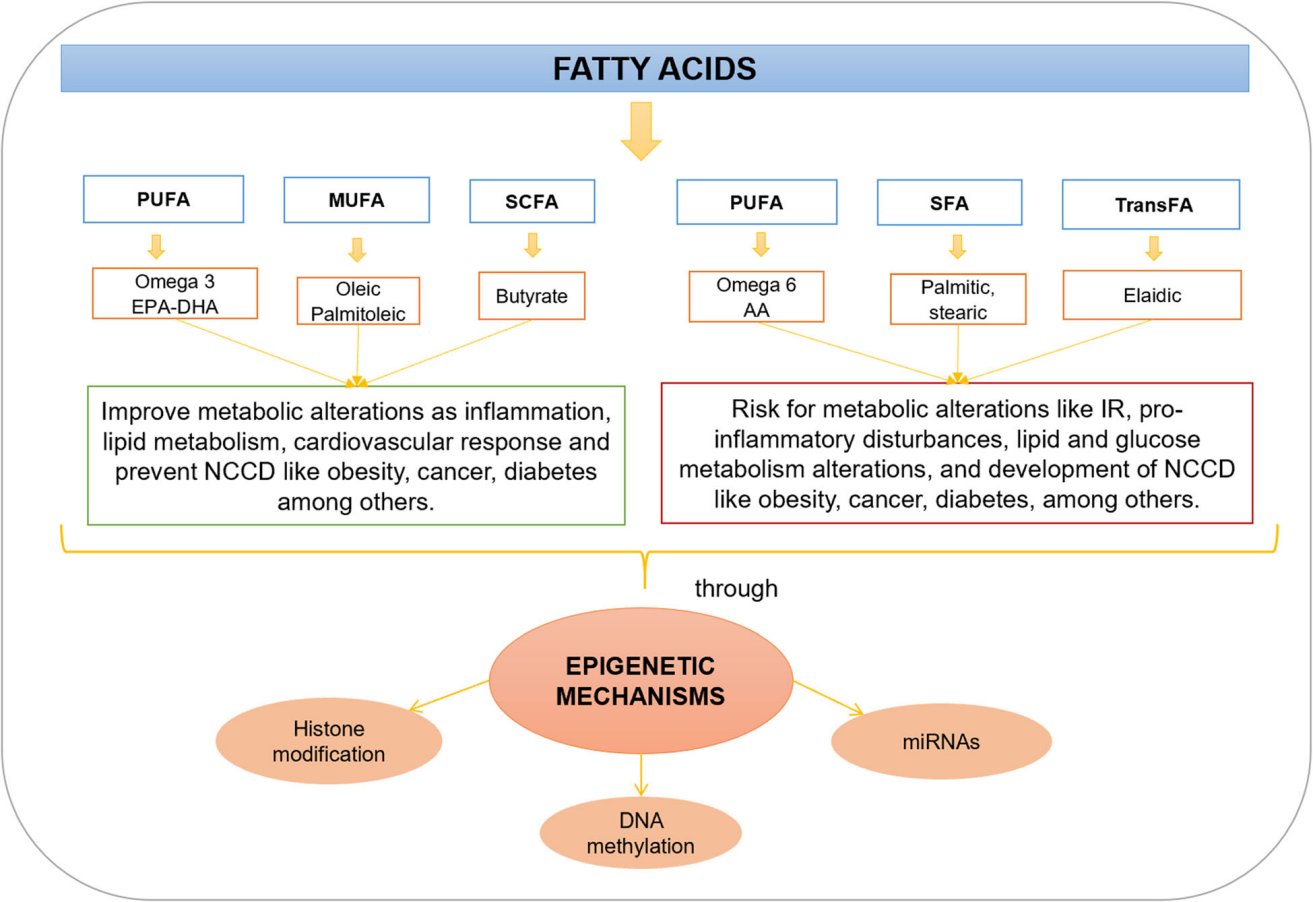
Summary of the main metabolic effects of fatty acids that can be mediated by epigenetic mechanisms. PUFA: Polyunstaturated fatty acids, MUFA: Monounsaturated fatty acids, SFA: Saturated fatty acids, SCFA: Short chain fatty acids, EPA: Eicosapentanoic acid, DHA: Docosahexanoic acid, AA: Arachidonic acid, NCCD: Non-comunicable cronic disease, miRNAS: Non-coding microRNAs, IR: Insulin Resitance

The existing results confirm that FA can influence DNA methylation (hyper or hypomethylation) as well as acetylation or deacetylation of histones, and miRNAs associated with the repression, or activation of genes. For example, n-3 PUFA (i.e., EPA-DHA), and MUFA (i.e., OA, palmitoleic) have been related to the prevention of metabolic alterations (lipid metabolism disturbances, inflammation, and IR) or chronic diseases (obesity, T2D, non-alcoholic fatty liver disease, cardiovascular risk and some types of cancer). On the other hand, n-6 PUFA, such as AA, SFA (stearic and palmitic), and TFA (elaidic acid), have been associated with the presence or development of obesity, T2D, inflammatory profile, atherosclerosis, hyperglycemia, IR, lipid alterations, lipotoxicity, dysregulation of lipid metabolism, and abnormal lipid accumulation (Fig. 2).

Concerning the effects of n-3 PUFA on the DNA methylation status, a possible mechanism that has been proposed is that n-3 PUFA can promote the conversion from C to 5mC conducted by DNA methyltransferases (DNMTs) by enhancing the expression of DNMTs and consequently influence DNA methylation [20]. Furthermore, a potential interaction between n-3 PUFA and MeCP2 (methyl CpG binding protein 2) has been proposed, mainly in promoter regions, and consequently could be associated with the regulation of gene expression [50]. Another possible mechanism by which n-3 PUFAs can affect methylation is that these FA are natural ligands of some transcriptional factors, such as PPAR*γ* [51]. In this context, it has been reported that interactions between PPARγ and fatty acids result in a decrease in cytokine expression [52], and in murine models, *Pparg* expression is modulated by DNA methylation in its promoter region [61]. However, more studies are needed to elucidate the role of FA in the regulation of epigenetic mechanisms in the context of metabolic alterations and chronic diseases. Regarding the other types of FA, a specific mechanism in which they could alter epigenetic landmarks, has not been described.

The intake and supplementation of different types of FA has demonstrated to have an effect on transgenerational epigenetic mechanisms (being DNA methylation the most studied). These effects are implicated in the pathogenic or protective role of FA and can be modulated during pregnancy and lactation, suggesting that they could be interesting therapeutic targets.

In this term the role of nutraceuticals as a potent effect on lipids regulation should be considered, and more investigations are necessary to elucidate the role of nutraceuticals depending of the individual genetic variability [63], and their possible effect on epigenetic modifications for finally encourage the management of metabolic diseases as an integrative treatment.

SCFA are especially interesting because they take part of a diet-microbiota-epigenetics axis. For example, butyrate is a potent non-competitive HDAC inhibitor that is implicated in the regulation of gene expression. However, more studies are necessary to understand the regulation of specific genes and consequently their metabolic effects, as well as to consider the integrative effect of other components like gut microbiota, because butyrate is mainly produced by gut microbes, so the interaction will be very important to understand the complete outcome [64].

The epigenetic and metabolic effects of the different types of FA depend on the dose and the model, but many examples demonstrate that they can modulate the epigenome. Nevertheless, more studies are necessary to clarify the specific genes and pathways that are affected by FA through epigenetic mechanisms and consider other nutritional components that have an effect on epigenetic landmarks, such as methyl donors (vitamin B_12_, folate, choline, betaine, methionine, serine, glycine, and histidine), vitamins (retinol, tocopherols, and ascorbate), and polyphenols (epigallocatechin 3-gallate, genistein, curcumin, resveratrol, and sulforaphane, among others).

## Supplementary information

**Additional file 1.** Search strategy and data extraction.

## Data Availability

Not applicable.

## Abbreviations

AA: Arachidonic acid
CVD: Cardiovascular Disease
DHA: Docosahexaenoic acid
DMRs: Differentially methylated regions
DNMTs: DNA methyltransferases
EA: Elaidic acid
EPA: Eicosapentaenoic
FA: Fatty acids
HDAC: Histone deacetylases
IR: Insulin Resistance
MBD2: Methyl-CpG-binding domain protein 2
MUFA: Monosaturated fatty acids
NaB: Sodium butyrate
NCCD: Non-comunicable chronic disease
NEFA: Non-esterified fatty acids
OA: Oleic acid
PUFA: Polyunsaturated fatty acids
SCFA: Short chain fatty acids
SFA: Saturated fatty acids
T2D: Type 2 Diabetes
TFA: Trans fatty acids
