# Supplementary material for: Fatty acids, epigenetic mechanisms and chronic diseases: a systematic review
Source: Lipids Health Dis. 2019 Oct 15;18:178. doi: 10.1186/s12944-019-1120-6 (PMC6792183; doi:10.1186/s12944-019-1120-6)
Supplement: Supplementary file 1 — Additional file 1. Search strategy and data extraction. [file 12944_2019_1120_MOESM1_ESM.docx]

**Supplementary material**

**Search strategy**

The search strategy combined terms related epigenetic and fatty acids, included in title and/or abstract. “fatty acids and epigenetic mechanisms “unsaturated fatty acids and epigenetic” “polyunsaturated fatty acids and epigenetic” “omega 3 and epigenetic” “saturated fatty acids and epigenetic” “trans fatty acids and epigenetic” “short chain fatty acids and epigenetic” “butyric acid and epigenetic” butyrate and epigenetic” “fatty acids and epigenomics” “unsaturated fatty acids and epigenomics” “polyunsaturated fatty acids and epigenomics” “omega 3 and epigenomics” “saturated fatty acids and epigenomics” “trans fatty acids and epigenomics” “short chain fatty acids and epigenomics” “butyric acid and epigenomics” butyrate and epigenomics” “fatty acids and DNA methylation” “unsaturated fatty acids and DNA methylation” “polyunsaturated fatty acids and DNA methylation” “omega 3 and DNA methylation” “saturated fatty acids and DNA methylation” “trans fatty acids and DNA methylation” “short chain fatty acids and DNA methylation ” “butyric acid and DNA methylation” butyrate and DNA methylation” “fatty acids and histone modification” “unsaturated fatty acids and histone modification” “polyunsaturated fatty acids and histone modification” “omega 3 and histone modification” “saturated fatty acids and histone modification” “trans fatty acids and histone modification” “short chain fatty acids and histone modification” “butyric acid and histone modification” butyrate and histone modification” “fatty acids and miRNAs” “unsaturated fatty acids and miRNAs” “polyunsaturated fatty acids and miRNAs” “omega 3 and miRNAs” “saturated fatty acids and miRNAs” “trans fatty acids and miRNAs” “short chain fatty acids and miRNAs” “butyric acid and miRNAs” butyrate and miRNAs”.

**Eligibility criteria**

Studies to be included either described first an association between epigenetic marks (global, site specific or genome-wide methylation of DNA, histone modifications or miRNAs changes), and some type of fatty acids (supplemented or intake through the diet in humans or animal models and in case of cells that were stimulated with some type of fatty acid) and the third criterion that were related with some illness or metabolic alteration (lipid dysregulation, inflammation, insulin resistance, lipotoxicity oxidative stress, adipogenesis, dyslipidemias, atherosclerosis, diabetes, obesity and cancer).

For the final selection two reviewers, screened the retrieved titles and abstracts and selected eligible studies. In case of disagreement, decision was made through consensus or consultation with a third independent reviewer. Full texts were retrieved for studies that satisfied all selection criteria.

**Data extraction**

A predesigned data collection form was realized by the authors to extract the relevant information from the included studies, two authors as independent extracted the date and then were contrasted. Any disagreements which cannot be resolved by discussion will be referred to a third reviewer who will act as an arbitrator.

Data were extracted in a format that include the study design, the type of FA, the dose and duration of follow up (for longitudinal studies), study model (human, animals or cells), the epigenetic mechanism (DNA methylation, histone modification or miRNAs), the type of epigenetic signature (hypomethylation, hypermethylation, increase or decrease of global methylation, hyperacetylation) and finally the metabolic effect.
